# Emergence of Atrial Fibrillation and Flutter in COVID-19 Patients: A Retrospective Cohort Study

**DOI:** 10.3390/healthcare12171682

**Published:** 2024-08-23

**Authors:** Tanzim Bhuiya, Paras P. Shah, Wing Hang Lau, Timothy Park, Rezwan F. Munshi, Ofek Hai, Roman Zeltser, Amgad N. Makaryus

**Affiliations:** 1 Department of Internal Medicine, Hospital of the University of Pennsylvania, Philadelphia, PA 191104, USA; 2 Department of Internal Medicine, Donald and Barbara Zucker School of Medicine at Hofstra/Northwell, Hempstead, NY 11549, USA; 3 Department of Cardiology, Nassau University Medical Center, East Meadow, NY 11554, USA; 4 Department of Cardiology, Donald and Barbara Zucker School of Medicine at Hofstra/Northwell, Hempstead, NY 11549, USA

**Keywords:** COVID-19, atrial fibrillation, atrial flutter, sepsis, elderly, arrhythmias

## Abstract

COVID-19 is associated with various cardiovascular complications, including arrhythmias. This study investigated the incidence of new-onset atrial fibrillation (AFB) and atrial flutter (AFL) in COVID-19 patients and identified potential risk factors. We conducted a retrospective cohort study at a tertiary-care safety-net community hospital including 647 patients diagnosed with COVID-19 from March 2020 to March 2021. Patients with a prior history of AFB or AFL were excluded. Data on demographics, clinical characteristics, and outcomes were collected and analyzed using chi-square tests, *t*-tests, and binary logistic regression. We found that 69 patients (10.66%) developed AFB or AFL, with 41 patients (6.34%) experiencing new-onset arrhythmias. The incidence rates for new-onset AFB and AFL were 5.4% and 0.9%, respectively. Older age (≥65 years) was significantly associated with new-onset AFB/AFL (OR: 5.43; 95% CI: 2.31–12.77; *p* < 0.001), as was the development of sepsis (OR: 2.73; 95% CI: 1.31–5.70; *p* = 0.008). No significant association was found with patient sex. Our findings indicate that new-onset atrial arrhythmias are a significant complication in COVID-19 patients, particularly among the elderly and those with sepsis. This highlights the need for targeted monitoring and management strategies to mitigate the burden of atrial arrhythmias in high-risk populations during COVID-19 infection.

## 1. Introduction

The first case of pneumonia caused by the severe acute respiratory syndrome coronavirus 2 (SARS-CoV-2) infection occurred in Wuhan City, Hubei Province, China in December 2019 [1]. Based on the large-scale community transmission, the World Health Organization declared COVID-19 a pandemic [2]. The clinical manifestations of COVID-19 vary widely, ranging from asymptomatic and, mild upper respiratory symptoms to acute respiratory distress syndrome (ARDS) and death [3]. Important clinical complications among patients hospitalized with the disease are cardiovascular manifestations. COVID-19 has been associated with a myriad of cardiac manifestations, including acute coronary syndromes, myocarditis, cardiomyopathies, arrhythmias, heart failure, and thromboembolic disease [4]. Up to 20–30% of patients hospitalized with COVID-19 showed signs of myocardial involvement manifested by elevated troponin levels [5]. These complications arise from mechanisms such as direct viral invasion of cardiac cells, systemic inflammation, and a hypercoagulable state [6].

Cardiac manifestations and arrhythmias represent a significant sequalae of COVID-19, with atrial arrhythmias emerging as the most prevalent [7,8,9]. Arrhythmias have been reported to occur in up to 16.7% of patients presenting with severe ARDS secondary to COVID-19 [10]. A study based at Rush University Medical Center in Chicago showed the rate of arrhythmias as 9.6% [11]. During the first peak of the pandemic, atrial fibrillation was the most common electrophysiology consult in patients with COVID-19 (31%) at Columbia University, and only 13% had a prior history of the disease [9]. Another study showed atrial fibrillation/flutter occurred in 22% of patients, and only 9% had a prior history [12]. Ventricular arrhythmias such as ventricular tachycardia and ventricular fibrillation were also seen, but much less commonly. Only seven percent of consults were for ventricular arrythmias in the Columbia study compared to 31% for ventricular arrhythmias. Bradyarrhythmias such as bradycardia and atrioventricular blocks were seen in 12% of arrythmias in patients with COVID-19 [9].

To minimize complications, awareness regarding what demographic and clinical factors elevate the risk of arrhythmias becomes imperative. This awareness helps guide stratification of resources, including medications, interventions, and heightened monitoring such as telemetry especially in resource-limited hospitals. This study aims to investigate the incidence of new-onset atrial fibrillation (AFB) and atrial flutter (AFL) among patients with COVID-19 within a tertiary-care safety-net community hospital and to identify potential risk factors associated with these atrial arrhythmias. The findings are intended to provide insights for targeted strategies in monitoring and intervention for at-risk patients diagnosed with COVID-19.

## 2. Methods

A retrospective cohort study at a tertiary-care hospital in New York state was performed on patients diagnosed with COVID-19 from March 2020 to March 2021. The study was approved by the Institutional Review Board and conducted in accordance with the tenets of the Declaration of Helsinki.

The number of COVID-19 patients diagnosed with new-onset AFB or AFL during their hospital course were evaluated. This was determined based on retrospective review of various sources, including admission assessment and plan, admission electrocardiogram (EKG), hospitalist progress note, cardiology consult/progress note, medical intensive care unit consult/progress note, and/or repeat EKG during the full hospital course. Patients with a prior history of AFB or AFL were then excluded from the analysis. This was determined based on review of the patient’s prior medical history on admission and progress notes, as well as review of the patient’s problem list and corresponding date of entry. Additional demographic and medical information for each patient was recorded, including age, race, sex, body mass index, history of hypertension, coronary artery disease, and diabetes mellitus. The diagnosis of sepsis was based on Sepsis-2 guidelines, and older age was defined as greater than 65 years. 

The groups with and without new-onset AFB/AFL were compared using chi-square tests for categorical characteristics and *t*-tests for continuous variables. Binary logistic regression, adjusted for race, sex, body mass index, and history of hypertension, coronary artery disease, diabetes mellitus, and chronic obstructive pulmonary disease, was performed to determine the association between new-onset AFB or AFL and patient characteristics such as older age and presence of sepsis. SPSS, Version 26 (IBM Inc., Armonk, NY, USA) was utilized for all analyses, and a 2-sided *p*-value ≤ 0.05 was considered statistically significant.

## 3. Results

**Figure 1:**
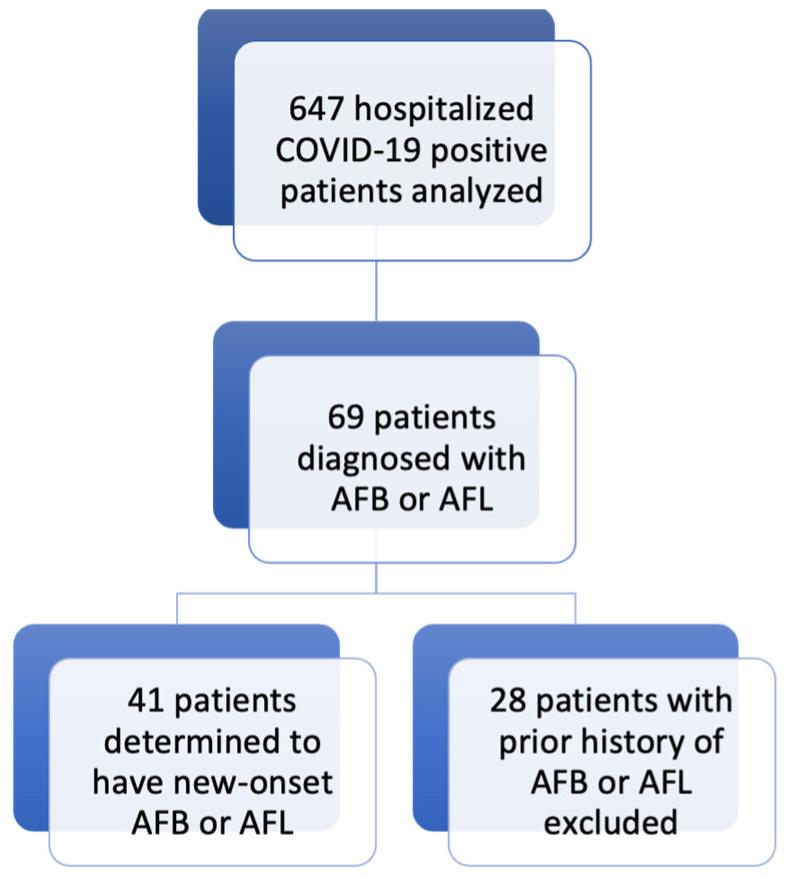
Flowchart of the selection process of the study cohort.

A total of 647 patients diagnosed with COVID-19 were included in this analysis (Table 1). Overall, 69 (10.7%) patients developed AFB or AFL during their hospital course, of which 28 were excluded due to a prior history of AFB or AFL in the medical record. This yielded 41 (6.34%) patients who developed new-onset AFB or AFL during a COVID-19 infection (Figure 1). A total of 35 of these patients (85.4%) had new-onset AFB, while 6 (14.6%) patients developed new-onset AFL. This is indicative of a 5.4% overall incidence of new-onset AFB and 0.9% overall incidence of new-onset AFL. 

Patients with new-onset AFB or AFL were significantly more likely to be elderly (χ^2^ = 21.930, df = 1, *p* < 0.001) and septic (χ^2^ = 10.074, df = 1, *p* = 0.002) as indicated by the chi-square test of independence. Patient sex was not associated with the development of new-onset AFB or AFL (χ^2^ = 0.300, df = 1, *p* = 0.584). Furthermore, an independent samples *t*-test demonstrated that the mean age of patients in the new-onset AFB/AFL group was significantly higher than that of patients who did not develop new-onset AFB/AFL (72.3 versus 57.1, respectively; T = 5.140, 95% CI: 9.46–21.17, *p* < 0.001). Among all COVID-19 patients, our multivariate logistic regression model, adjusted for race, sex, body mass index, and history of hypertension, coronary artery disease, diabetes mellitus, and chronic obstructive pulmonary disease, revealed that the development of sepsis (OR: 2.73; 95th% CI: 1.31–5.70; *p* = 0.008) and age ≥ 65 years (OR: 5.43; 95th% CI: 2.31–12.77; *p* < 0.001) were associated with significantly higher odds of developing new-onset AFB or AFL (Table 2). 

## 4. Discussion

Our results showing age and sepsis as risk factors for arrhythmia are in line with the results found in other studies. The 2023 ACC/AHA/ACCP/HRS guideline recognizes sepsis and advancing age as risk factors for atrial fibrillation [13]. With every five-year increase in age, the risk of incident AF increases (HR of 1.43–1.66). Severe sepsis increases the risk of atrial fibrillation (OR of 6.82) [14,15,16]. A retrospective study performed at New York-Presbyterian/Weill Cornell Medicine and New York-Presbyterian/Lower Manhattan Hospital showed age, male sex, prior history of atrial fibrillation, and hypoxia on presentation to be associated with the risk of any arrhythmia [17]. The significance of these findings cannot be overstated. Atrial fibrillation (AFB) is associated with a 1.5- to 2-fold increased risk of mortality. Additionally, studies indicate that AFB is linked to a higher likelihood of other adverse outcomes, including a 1.4-fold increase in the risk of stroke, a 1.5-fold increase in the risk of cognitive impairment, a 1.5-fold increase in the risk of myocardial infarction (MI), a 2-fold increase in the risk of sudden cardiac death, and a 5-fold increase in the risk of heart failure [18,19,20,21]. In the Medicare population, the most frequent outcome within five years following an AFB diagnosis was death, occurring in 19.5% of cases at one year and 48.8% at five years [22]. The substantial morbidity and mortality associated with AFB underscore the critical importance of identifying individuals at risk and implementing appropriate management strategies. For example, a study by Sonaglioni et al. showed increased comorbidity burden, elevated serum levels of inflammatory biomarkers such as the neutrophil-to-lymphocyte ratio (NLR), and undertreatment with ACE inhibitors (ACEis) and angiotensin II receptor blockers (ARBs) contributed to the development of atrial arrhythmias and unfavorable outcomes in COVID-19 patients. The study highlighted that higher NLR levels were significantly associated with a higher risk of atrial arrhythmias in this population. Furthermore, the potential underutilization of ACEis and ARBs may exacerbate the susceptibility to arrhythmias by failing to counteract the renin–angiotensin–aldosterone system’s (RAAS) hyperactivation, which plays a critical role in the inflammatory response and cardiovascular complications observed in COVID-19 patients [23].

While the COVID-19 pandemic has subsided, the relevance of understanding its cardiac implications remains significant, particularly when compared with other respiratory infections. Previous research, such as that by Kochi et al., has demonstrated that other respiratory syndromes, including severe acute respiratory syndrome-coronavirus 2 (SARS-CoV-2), H1N1 Influenza, and Middle East respiratory syndrome-coronavirus (MERS-CoV), also led to substantial cardiovascular complications, including arrhythmias. This suggests that viral-induced cardiac injury may be a common pathway across various respiratory infections [24].

There are many proposed mechanisms as to the arrhythmogenicity of COVID-19. There are a variety of different methods of injury to the myocardium in addition to extracardiac processes that can exacerbate arrhythmias in at-risk patients. Between 19.7 and 27.8% of patients with COVID-19 sustain a myocardial injury as seen as elevated cardiac troponin levels [25]. The presence of myocardial injury increases the risk of arrhythmias from 1.5% to 17.3% [26]. Viral infection can increase metabolic demand and deplete cardiac reserve causing those with cardiovascular disease to become unstable [27]. Additionally, hypoxia from the disease can cause cell damage and facilitate deranged cell depolarizations and temporal alterations during action potentials throughout the cardiac cycle. Hypoxia can also increase extracellular potassium, which decreases depolarization threshold and accelerates electrical conduction [28]. COVID-19 has also been shown to cause myocarditis [29]. Myocarditis can cause arrhythmia through direct cell damage, ion channel impairment, and gap junction dysfunction [30]. Electrolyte disturbances have been reported in a case series in patients with COVID-19 infection and is a known risk factor for arrhythmias [25]. Similarly, sepsis can cause atrial fibrillation in patients through profound systemic inflammation, oxidative stress, and myocardial injury. This inflammatory cytokine surge in sepsis disrupts electrical activity of the heart putting patients at risk of arrhythmias [31]. Age is another significant factor, as age-related structural changes, such as fibrosis and atrial dilation, increase the vulnerability of the myocardium to abnormal electrical impulses, further elevating the risk of arrhythmias [32]. 

Several studies have evaluated the incidence of arrhythmias in patients with severe COVID-19 disease. One early meta-analysis pooled data from over 1500 patients and five studies and found that patients severely ill with COVID-19 pneumonia were nearly 18-times more likely to develop arrhythmias when compared to patients with non-severe COVID-19. However, this study did not specifically evaluate for new-onset arrhythmias, indicating the possibility of confounding underlying arrhythmias. They also grouped all arrhythmias into one, while we focused specifically on new-onset AFB and AFL [33]. A more recent meta-analysis focusing on new-onset atrial fibrillation, which included nearly 20 million patients, found that the pooled incidence of new-onset AF was 2.6%, which is slightly lower than what we found in our study (5.4%) [34]. One possible reason for this discrepancy could be inconsistent recording of prior AFB or AFL in the electronic medical record. Interestingly, patients who recovered from COVID-19 continued to have a higher risk of incident AFB when compared to non-infected patients, indicating that the effects of infection may persist following recovery [34]. Other studies have described this effect as long COVID, which has been associated with various cardiovascular abnormalities, including myocardial inflammation, myocardial infarction, and arrhythmias, even after recovery from acute illness [35]. 

The pillars of AF management according to The 2023 ACC/AHA/ACCP/HRS Guideline is to minimize stroke risk and optimize modifiable risk factors and symptom management through rate and rhythm controls. However, there are some specific considerations in COVID-19 patients; for example, given the risk of respiratory bronchoconstriction in COVID-19 patients on beta blockers, the use of calcium channel blockers has been recommended [36]. Importantly, there have been many documented drug–drug interactions in these clinically complex situations, and there continues to be a lack of clear recommendations on managing atrial arrhythmias in COVID-19 patients [37]. The use of prophylactic antiarrhythmic medication has previously been suggested for certain high-risk patients infected with COVID-19 [38]. However, this is controversial given that various antiarrhythmic drugs and even COVID-19 therapies are associated with potentially life-threatening QT prolongation [39,40]. 

## 5. Limitations

While this study benefits from a large, diverse cohort of patients within a tertiary-care safety-net community hospital, limitations must be acknowledged. Firstly, the retrospective design inherently introduces potential biases and limits the ability to establish causality between COVID-19 and the development of new-onset atrial fibrillation (AFB) or atrial flutter (AFL). Additionally, it remains challenging to discern whether these arrhythmias are a direct result of COVID-19 infection or a consequence of pharmacological treatments, including medications that may have proarrhythmic effects, such as QT-prolonging agents. Unfortunately, due to the retrospective nature of this study, we were unable to collect detailed data on the use of antiarrhythmic drugs or COVID-19-specific treatments, which may have influenced the incidence of AFB/AF thus restricting our ability to fully assess the impact of these factors on the observed outcomes. Future studies should aim to include treatment data to better elucidate the relationship between COVID-19 management strategies and the occurrence of atrial arrhythmias. Despite these limitations, our study provides important insights into the arrhythmogenic potential of COVID-19 and underscores the need for continued research in this area.

## 6. Conclusions

Our study highlights the burden of atrial arrhythmias, specifically atrial fibrillation and atrial flutter, as a consequence of COVID-19 infection. Our results are consistent with other studies in highlighting sepsis and advanced age as risk factors for developing arrhythmias secondary to COVID-19. Our findings highlight the critical need for vigilant monitoring of at-risk patients to safeguard this already vulnerable population from the onset of atrial arrhythmias.

The parallels between COVID-19 and these earlier respiratory syndromes highlight the broader implications of our findings. By comparing the cardiac outcomes observed in COVID-19 with those in SARS, H1N1, and MERS, we emphasize the necessity of continued research into the cardiovascular effects of severe viral infections. This understanding is not only critical for managing COVID-19 patients but also for improving outcomes in future viral outbreaks that may pose similar risks.

## Figures and Tables

**Table 1:** Clinical features and characteristics of the study population. Age is presented as mean ± standard deviation (SD), and other variables are presented as counts with percentages.

Variable	No. (%) or Mean
**Age**	**Mean, Years**
Overall Population	58.1 ± 18.8
Population With AFB/AFL	72.3 ± 12.1
Population Without AFB/AFL	57.1 ± 18.8
**Sex**	**N (%)**
Male	384 (59.4%)
Female	267 (40.6%)
**Race/Ethnicity**	**N (%)**
White	243 (37.6%)
Black/African American	133 (20.6%)
Hispanic/Latino	42 (6.5%)
Eastern Asian	23 (3.6%)
South Asian	191 (29.5%)
Native Hawaiian/Pacific Islander	9 (1.4%)
American Indian/Alaskan	5 (0.8%)

**Table 2:** Multivariate logistic regression analysis of sepsis, age, gender, and other risk factors for new-onset atrial fibrillation and flutter in COVID-19 patients: Odds ratios (ORs), 95% confidence intervals (CIs), and *p*-values. (†) indicates insufficient sample size for statistical computation. Significant *p*-values (*p* < 0.05) are marked with an asterisk (*).

Variable	Odds Ratio	95% CI Lower	95% CI Upper	Significance (*p* Value)
Sepsis (0—no sepsis, 1—sepsis)	2.723	1.31	5.70	0.01 *
Age ≥ 65 (0 < 65, 1 ≥ 65)	5.43	2.31	12.77	<0.001 *
Gender (0 = Male, 1 = Female)	1.50	0.72	3.09	0.28
Diabetes Mellitus (0 = No DM, 1 = DM)	1.00	0.46	2.20	0.99
BMI Categories (0—underweight = <18.5; 1—normal = 18.5–24.9; 2—overweight = 25–29.9; 3—obese class I—30–34.9; 4—obese class II—35–39.9; 5—morbid obesity—equal to or more > 40)				
Normal (18.5–24.9)	0.53	0.05	5.34	0.59
Overweight (25–29.9)	0.65	0.07	6.28	0.71
Obese Class I (30–34.9)	1.18	0.12	12.06	0.89
Obese Class II (35–39.9)	0.55	0.04	7.57	0.65
Morbid Obesity (≥40)	1.53	.12	19.23	0.74
Hypertension (0—no hypertension, 1—hypertension)	1.54	0.66	3.59	0.32
CAD (0 = no CAD, 1 = CAD)	0.87	0.32	2.35	0.78
Race 0 = White 1 = Black/African American 2 = Hispanic/Latino 3 = Eastern Asian 4 = South Asian 5 = Native Hawaiian/Pacific Islander 6 = American Indian/Alaskan				
Black/African American	0.27	0.08	0.96	0.16
Hispanic/Latino	1.67	0.52	5.26	0.12
Eastern Asian	4.88	0.86	27.75	0.21
South Asian	1.2	0.52	2.78	0.71
Native Hawaiian/Pacific Islander	†	†	†	†
American Indian/Alaskan	†	†	†	†

## Data Availability

Data are contained within the article.

## References

[1] Shi Y., Wang G., Cai X.-P., Deng J.-W., Zheng L., Zhu H.-H., Zheng M., Yang B., Chen Z. (2020). An overview of COVID-19. J. Zhejiang Univ. Sci. B.

[2] Jee Y. (2020). WHO International Health Regulations Emergency Committee for the COVID-19 outbreak. Epidemiol. Health.

[3] Zhou F., Yu T., Du R., Fan G., Liu Y., Liu Z., Xiang J., Wang Y., Song B., Gu X. (2020). Clinical course and risk factors for mortality of adult inpatients with COVID-19 in Wuhan, China: A retrospective cohort study. Lancet.

[4] Keri V.C., Hooda A., Kodan P., RL B., Jorwal P., Wig N. (2021). Intricate interplay between Covid-19 and cardiovascular diseases. Rev. Med. Virol..

[5] Mitrani R.D., Dabas N., Goldberger J.J. (2020). COVID-19 cardiac injury: Implications for long-term surveillance and outcomes in survivors. Heart Rhythm..

[6] Zaman S., MacIsaac A.I., Jennings G.L., Schlaich M.P., Inglis S.C., Arnold R., Kumar S., Thomas L., Wahi S., Lo S. (2020). Cardiovascular disease and COVID-19: Australian and New Zealand consensus statement. Med. J. Aust..

[7] Zylla M.M., Merle U., Vey J.A., Korosoglou G., Hofmann E., Müller M., Herth F., Schmidt W., Blessing E., Göggelmann C. (2021). Predictors and Prognostic Implications of Cardiac Arrhythmias in Patients Hospitalized for COVID-19. J. Clin. Med..

[8] Denegri A., Sola M., Morelli M., Farioli F., Alberto T., D’Arienzo M., Savorani F., Pezzuto G.S., Boriani G., Szarpak L. (2022). Arrhythmias in COVID-19/SARS-CoV-2 Pneumonia Infection: Prevalence and Implication for Outcomes. J. Clin. Med..

[9] Berman J.P., Abrams M.P., Kushnir A., Rubin G.A., Ehlert F., Biviano A., Morrow J.P., Dizon J., Wan E.Y., Yarmohammadi H. (2020). Cardiac electrophysiology consultative experience at the epicenter of the COVID-19 pandemic in the United States. Indian Pacing Electrophysiol. J..

[10] Wang D., Hu B., Hu C., Zhu F., Liu X., Zhang J., Wang B., Xiang H., Cheng Z., Xiong Y. (2020). Clinical Characteristics of 138 Hospitalized Patients With 2019 Novel Coronavirus–Infected Pneumonia in Wuhan, China. JAMA.

[11] Gottlieb M., Sansom S., Frankenberger C., Ward E., Hota B. (2020). Clinical Course and Factors Associated with Hospitalization and Critical Illness Among COVID-19 Patients in Chicago, Illinois. Acad. Emerg. Med..

[12] Bertini M., Ferrari R., Guardigli G., Malagù M., Vitali F., Zucchetti O., D’Aniello E., Volta C.A., Cimaglia P., Piovaccari G. (2020). Electrocardiographic features of 431 consecutive, critically ill COVID-19 patients: An insight into the mechanisms of cardiac involvement. EP Eur..

[13] Joglar J.A., Chung M.K., Armbruster A.L., Benjamin E.J., Chyou J.Y., Cronin E.M., Deswal A., Eckhardt L.L., Goldberger Z.D., Gopinathannair R. (2024). 2023 ACC/AHA/ACCP/HRS Guideline for the Diagnosis and Management of Atrial Fibrillation: A Report of the American College of Cardiology/American Heart Association Joint Committee on Clinical Practice Guidelines. Circulation.

[14] Alonso A., Krijthe B.P., Aspelund T., Stepas K.A., Pencina M.J., Moser C.B., Sinner M.F., Sotoodehnia N., Fontes J.D., Janssens A.C.J.W. (2013). Simple Risk Model Predicts Incidence of Atrial Fibrillation in a Racially and Geographically Diverse Population: The CHARGE-AF Consortium. J. Am. Heart Assoc..

[15] Allan V., Honarbakhsh S., Casas J.P., Wallace J., Hunter R., Schilling R., Perel P., Morley K., Banerjee A., Hemingway H. (2017). Are cardiovascular risk factors also associated with the incidence of atrial fibrillation? A systematic review and field synopsis of 23 factors in 32 population-based cohorts of 20 million participants. Thromb. Haemost..

[16] Walkey A.J., Wiener R.S., Ghobrial J.M., Curtis L.H., Benjamin E.J. (2011). Incident Stroke and Mortality Associated With New-Onset Atrial Fibrillation in Patients Hospitalized With Severe Sepsis. JAMA.

[17] Peltzer B., Manocha K.K., Ying X., Kirzner J., Ip J.E., Thomas G., Liu C.F., Markowitz S.M., Lerman B.B., Safford M.M. (2020). Arrhythmic Complications of Patients Hospitalized With COVID-19. Circ. Arrhythmia Electrophysiol..

[18] Odutayo A., Wong C.X., Hsiao A.J., Hopewell S., Altman D.G., Emdin C.A. (2016). Atrial fibrillation and risks of cardiovascular disease, renal disease, and death: Systematic review and meta-analysis. BMJ.

[19] Papanastasiou C.A., Theochari C.A., Zareifopoulos N., Arfaras-Melainis A., Giannakoulas G., Karamitsos T.D., Palaiodimos L., Ntaios G., Avgerinos K.I., Kapogiannis D. (2021). Atrial Fibrillation Is Associated with Cognitive Impairment, All-Cause Dementia, Vascular Dementia, and Alzheimer’s Disease: A Systematic Review and Meta-Analysis. J. Gen. Intern. Med..

[20] Ruddox V., Sandven I., Munkhaugen J., Skattebu J., Edvardsen T., Otterstad J.E. (2017). Atrial fibrillation and the risk for myocardial infarction, all-cause mortality and heart failure: A systematic review and meta-analysis. Eur. J. Prev. Cardiol..

[21] Rattanawong P., Upala S., Riangwiwat T., Jaruvongvanich V., Sanguankeo A., Vutthikraivit W., Chung E.H. (2018). Atrial fibrillation is associated with sudden cardiac death: A systematic review and meta-analysis. J. Interv. Card. Electrophysiol..

[22] Walkey A.J., Greiner M.A., Heckbert S.R., Jensen P.N., Piccini J.P., Sinner M.F., Curtis L.H., Benjamin E.J. (2013). Atrial fibrillation among Medicare beneficiaries hospitalized with sepsis: Incidence and risk factors. Am. Heart. J..

[23] Sonaglioni A., Lombardo M., Albini A., Noonan D.M., Re M., Cassandro R., Elia D., Caminati A., Nicolosi G.L., Harari S. (2022). Charlson comorbidity index, neutrophil-to-lymphocyte ratio and undertreatment with renin-angiotensin-aldosterone system inhibitors predict in-hospital mortality of hospitalized COVID-19 patients during the omicron dominant period. Front. Immunol..

[24] Kochi A.N., Tagliari A.P., Forleo G.B., Fassini G.M., Tondo C. (2020). Cardiac and arrhythmic complications in patients with COVID-19. J. Cardiovasc. Electrophysiol..

[25] Shi S., Qin M., Shen B., Cai Y., Liu T., Yang F., Gong W., Liu X., Liang J., Zhao Q. (2020). Association of Cardiac Injury with Mortality in Hospitalized Patients With COVID-19 in Wuhan, China. JAMA Cardiol..

[26] Alblaihed L., Brady W.J., Al-Salamah T., Mattu A. (2023). Dysrhythmias associated with COVID-19: Review and management considerations. Am. J. Emerg. Med..

[27] Xiong T.Y., Redwood S., Prendergast B., Chen M. (2020). Coronaviruses and the cardiovascular system: Acute and long-term implications. Eur. Heart J..

[28] Lazzerini P.E., Boutjdir M., Capecchi P.L. (2020). COVID-19, Arrhythmic Risk, and Inflammation. Circulation.

[29] Guo T., Fan Y., Chen M., Wu X., Zhang L., He T., Wang H., Wan J., Wang X., Lu Z. (2020). Cardiovascular Implications of Fatal Outcomes of Patients with Coronavirus Disease 2019 (COVID-19). JAMA Cardiol..

[30] Dherange P., Lang J., Qian P., Oberfeld B., Sauer W.H., Koplan B., Tedrow U. (2020). Arrhythmias and COVID-19. JACC Clin. Electrophysiol..

[31] Kuipers S., Klein Klouwenberg P.M., Cremer O.L. (2014). Incidence, risk factors and outcomes of new-onset atrial fibrillation in patients with sepsis: A systematic review. Crit. Care.

[32] Lin Y.-K., Chen Y.-A., Lee T.-I., Chen Y.-C., Chen S.-A., Chen Y.-J. (2018). Aging Modulates the Substrate and Triggers Remodeling in Atrial Fibrillation. Circ. J..

[33] Wen W., Zhang H., Zhou M., Cheng Y., Ye L., Chen J., Wang M., Feng Z. (2020). Arrhythmia in patients with severe coronavirus disease (COVID-19): A meta-analysis. Eur. Rev. Med. Pharmacol. Sci..

[34] Zuin M., Ojeda-Fernández L., Torrigiani G., Bertini M. (2024). Risk of Incident Atrial Fibrillation after COVID-19 Infection: A Systematic Review and Meta-Analysis. Heart Rhythm..

[35] Raman B., Bluemke D.A., Lüscher T.F., Neubauer S. (2022). Long COVID: Post-acute sequelae of COVID-19 with a cardiovascular focus. Eur. Heart J..

[36] Bhatla A., Mayer M.M., Adusumalli S., Hyman M.C., Oh E., Tierney A., Moss J., Chahal A.A., Anesi G., Denduluri S. (2020). COVID-19 and cardiac arrhythmias. Heart Rhythm..

[37] Rattanawong P., Shen W., El Masry H., Sorajja D., Srivathsan K., Valverde A., Scott L.R. (2020). Guidance on Short-Term Management of Atrial Fibrillation in Coronavirus Disease 2019. J. Am. Heart Assoc..

[38] Lavelle M.P., Desai A.D., Wan E.Y. (2022). Arrhythmias in the COVID-19 patient. Heart Rhythm. O_2_.

[39] Rubin G.A., Desai A.D., Chai Z., Wang A., Chen Q., Wang A.S., Kemal C., Baksh H., Biviano A., Dizon J.M. (2021). Cardiac Corrected QT Interval Changes Among Patients Treated for COVID-19 Infection During the Early Phase of the Pandemic. JAMA Netw. Open.

[40] Manolis A.S., Manolis A.A., Manolis T.A., Apostolopoulos E.J., Papatheou D., Melita H. (2020). COVID-19 infection and cardiac arrhythmias. Trends Cardiovasc. Med..

